# Hydrothermal-induced changes in the gel properties of Mung bean proteins and their effect on the cooking quality of developed compound noodles

**DOI:** 10.3389/fnut.2022.957487

**Published:** 2022-08-04

**Authors:** Jingjing Diao, Yang Tao, Hongsheng Chen, Dongjie Zhang, Changyuan Wang

**Affiliations:** ^1^National Coarse Cereals Engineering Research Center, Heilongjiang Bayi Agricultural University, Daqing, China; ^2^Daqing Center of Inspection and Testing for Rural Affairs Agricultural Products and Processed Products, Ministry of Agriculture and Rural Affairs, Heilongjiang Bayi Agricultural University, Daqing, China; ^3^College of Food Science, Heilongjiang Bayi Agricultural University, Daqing, China

**Keywords:** hydrothermal treatment, Mung bean protein, modified Mung bean protein, gel properties, cooking quality

## Abstract

Mung bean proteins (MBPs) are highly nutritious food ingredients, but their lack of gluten limits their use in staple foods such as noodles. In this study, MBPs were modified by hydrothermal treatment, and their gel properties and the major structural changes were analyzed at different heating temperatures (25, 65, 75, 85, 95, and 105°C), moisture contents (0, 15, 20, 25, 30, and 35%), and hydrothermal treatment times (0, 15, 30, 45, 60, and 75 min). Thereafter, the modified MBPs (MMBPs) were added to wheat noodles at substitution levels of 3, 6, and 9% to evaluate their effect on the quality of the noodles. The results showed that the hydrothermal treatment significantly improved the gel properties and water absorption capacity of the MBPs and slightly increased their disulfide bond content. When MBPs with a 25% moisture content were heated at 85°C for 60 min, their gel properties notably improved, and their structural changes were maximal. The structural changes revealed that the MBP molecule formed a macromolecular polymer because a significant protein band appeared at about 66.2 kDa. Secondary structure and microstructure analyses revealed that the MBP structure was significantly damaged and that the β-sheet structure increased because of changes in the degree of aggregation between the protein molecules. Compared to the untreated MBPs, the MMBPs significantly improved the cooking quality and texture properties of the noodles, and the addition amount reached more than 6%, whereas that of the untreated MBPs was less than 3%. At this time, the cooking loss and the broken rate of the untreated MBPs group were about 2 times higher than that of the 6% MMBP-treated group. An analysis of changes in the water distribution, rheological properties, and microstructure revealed that intermolecular cross-linking occurred between the MMBPs and wheat dough, which improved the quality of the MMBP-treated noodles. The findings demonstrated that the MMBPs obtained by hydrothermal treatment had a positive effect on the wheat dough properties and noodle quality. These results provide a technical foundation for incorporating novel protein supplements into staple foods.

## Introduction

Noodles are a traditional and popular staple food in China, and they are mainly made of wheat and other grains. Wheat flour has a protein content of 12–15%, but lysine is the first limiting amino acid in its protein composition, making it unable to completely satisfy the requirement of essential amino acids (1). People are becoming increasingly aware of the importance of consuming healthy food as the prevalence of non-communicable nutritional diseases (such as hypertension, hyperlipidemia, and coronary heart disease) increases, and consumer demand for healthy food is also growing rapidly (2–5). Since the middle of the twentieth century, researchers have used food fortification with essential nutrients to improve nutritional deficiencies (6). Tazrart et al. indicated that adding legumes to grains could compensate for lysine deficiency in grains and methionine deficiency in beans, resulting in amino acid complementarity (7). These characteristics and market demand considerably enhance the value of legume proteins as potential ingredients.

Mung bean proteins (MBPs) are important high-quality plant-derived proteins with a high essential amino acid content (43.5% of the total amino acid content), and their nutritional score exceeds the recommended value of Food and Agriculture Organization (FAO)/World Health Organization (WHO) (8). Lysine, tyrosine, phenylalanine, and leucine are particularly abundant in MBPs (9). MBPs have been reported to have many beneficial physiological effects, such as improving glycolipid metabolism, preventing and controlling non-alcoholic fatty liver disease, and regulating antioxidant enzyme activity (10–12). However, MBPs are mostly used for producing protein beverages, emulsion stabilizers, etc., because they lack gluten, which limits their application in staple foods such as noodles. Numerous studies have verified that modification treatments such as high static pressure modification, extrusion and steam explosion, ultrasonic acid digestion, and hydrothermal treatment broaden the application areas of MBPs, soybean, and wheat bran (9, 13–16). In particular, hydrothermal modification treatment, which is the most common processing treatment in the food industry, has been proven to be very effective in modifying proteins. Yang et al. demonstrated that thermal modification treatment (95°C for 30 min) improved the gel properties of soybean proteins (17). Yan et al. discovered that quinoa protein gels produced in a water bath at 80–95°C had high chewiness, elasticity, cohesion, hardness, and adhesion (16). Some studies have also confirmed that the noodles made by mixing edible legume protein (peas, broad beans, soybeans, etc.) with wheat flour not only endows the noodles with higher protein content, but also significantly improves their taste, palatability and cooking characteristics (18–20). Gel properties are important for maintaining the quality of noodles, and improving the gel properties of MBPs will be an effective strategy to broaden its application in noodles. The objective of this study was to improve the gel properties of MBPs using a hydrothermal modification method based on gel property indicators such as gel strength, water absorption capacity, and disulfide bond content. Thereafter, the modified MBPs (MMBPs) obtained via hydrothermal treatment were added to noodles, and the effect of the gelled MBPs on the noodle quality was analyzed.

## Materials and methods

### Materials

Wheat flour (protein content 15%, w/w) and MBPs (protein content 92%, w/w) were purchased from a local market in Daqing, China. Additionally, β-Mercaptoethanol (β-ME) and sodium dodecyl sulfate (SDS) were obtained from Solarbio Co. (Beijing, China).

### Hydrothermal treatment of the Mung bean proteins

The MBPs were hydrothermally treated using the method described by Yan (16). MBP powder was laid flat on a tray and evenly sprayed with certain amounts of water (0, 15, 20, 25, 30, and 35%). Subsequently, they were sealed with cling film, placed in a blast drying oven (Yiheng Instruments Co., Shanghai, China), and heated at 25, 65, 75, 85, 95, and 105°C for 0, 15, 30, 45, 60, and 75 min, respectively. The hydrothermally treated MBPs were cooled to room temperature.

### Disulfide bond contents

The disulfide bond content in the control and hydrothermally treated MBP sample was measured using the total sulfhydryl group content and free sulfhydryl group content, as outlined by Ellman (21). In this experiment, 75 mg of the sample was dissolved into 10 mL of Tris-glycine buffer (8 mol/L), and then 1.0 mL of the protein solution was pipetted into 4.0 mL of Tris-glycine buffer and 0.05 mL Ellman’s reagent. The solution was rapidly mixed before being left at room temperature for 5 min. The free sulfhydryl content was measured at 412 nm. The total sulfhydryl content in the MBP samples was determined using the following method: 1 mL of the protein solution was pipetted into 0.05 mL of β-ME and 4.0 mL of Tris-glycine buffer, and the solution was mixed and kept at room temperature for 1 h. Next, 10 mL of 12% trichloroacetic acid was added and allowed to react for another 1 h. Thereafter, the protein solution was centrifuged at 4,000 r/min for 10 min. Subsequently, 1.0 mg of the protein precipitate was dissolved in 4.0 mL of Tris-glycine buffer and 0.04 mL of Ellman’s reagent. After rapid mixing, the solution was kept at room temperature for 5 min and measured at 412 nm.

### Gel strength

Texture profile analysis (TPA; TA. XT plus texture analyzer, Shanghai, China) was used to measure the gel strength according to a method described by Yang et al. (17). Each protein sample was diluted in deionized water to 20%, placed in a water bath, and heated at 80°C for 30 min before being cooled to room temperature and stored overnight at 4°C. The protein gel samples were analyzed by TPA, and the determination parameters were as follows: P/0.5 probe model, 1.0 mm/s probe running speed, 0.5 mm/s puncture running speed, 10 mm/s return speed, 5 mm puncture distance, and 1 g trigger type.

### Water absorption

The protein sample (1.0 g) was mixed with 20 g of deionized water and centrifuged at 4,500 r/min for 10 min after standing for 80 min at room temperature. The supernatant was weighed and used to calculate the adsorbed water content as a percentage of the protein content.

### Electrophoresis

The method of Zhang et al. (22) was used to perform SDS–polyacrylamide gel electrophoresis (SDS–PAGE). Here, 1 mL of the protein sample (0.5 mg/mL) was mixed with 1 mL of buffer (containing 0.25 mol/L of Tris–HCl, 0.5% bromophenol blue, 10% SDS, 50% glycerin, and 5% β-ME) and heated in boiling water for 5 min to denature the protein. The stacking and separating gels consisted of 5 and 12% acrylamide, respectively. Aliquots of 40 μL of protein per lane were loaded onto the stacking gels, and electrophoresis (DYY-8C, Beijing, China) was performed (laminated gel voltage: 80 mV; separation gel voltage: 120 mV). When the protein band reached the bottom of the electrophoresis machine, the gel was stained with Coomassie brilliant blue for 30 min and subsequently destained in a mixed solution with 5 w/v% methanol and 7.5 w/v% acetic acid until the band edge became colorless.

### Fourier-transform infrared analysis

Fourier-transform infrared (FTIR) spectroscopy was used to identify changes in the protein structure of the control and hydrothermally treated MBPs according to the method proposed by Zhang et al. (22). The FTIR spectra of the samples were recorded by scanning the MBP solution at a wavelength range of 400–4,000 cm^–1^ using a MAGNA-IR560 FTIR instrument (Thermo Nicolet Co., Wis. American).

### Modified Mung bean proteins-treated noodle preparation

The hydrothermally treated MMBPs were mixed with 3, 6, and 9% wheat flour (human daily protein intake of 68 g). Thereafter, 100 g of the mixed flour and 44% water were mixed in a kneading machine (ODA-5HM1, Beijing, China) for 10 min at medium speed to make a dough, which was then allowed to rest for 25 min in sealed containers at room temperature. The roll gap was adjusted to 2 mm, and the sheeted dough was doubled over and passed through the same gap five times. Thereafter, the roll gap was reduced to 1 mm, and the dough was cut into 2 mm wide noodles.

### Cooking quality

Cooking loss was determined using the method of Zhao et al. (23). Noodles (20 strips) were cooked in 100 mL of boiling tap water for 9 min and subsequently drained before drying the cooking water in an air oven at 105°C. The residue was weighed, and the results were expressed as a proportion of the uncooked dried noodles.

The broken rate of cooked noodles was determined using the same method described by Zhao et al. (23). Noodles (20 strips) were cooked in 100 mL of boiling tap water for 9 min. The cooked noodles were then removed, and the number of unbroken noodles was counted immediately.

Noodles (20 strips) were cooked in 100 mL of boiling tap water for 9 min, then removed and weighed. Water absorption capacity was expressed as the ratio of the weight of the cooked noodles to the weight of the uncooked noodles.

### Noodle texture analysis

The method of Zhao et al. (23) was used to perform a TPA on the cooked noodles. Noodles were cooked until done, placed in cold water for 2 min, and then drained for 30 s. The texture properties of the noodles were analyzed using a TA. XTplus texture analyzer (Ruibin Co., Shanghai, China). The TPA parameters for the cooked noodles were set as follows: P50 probe model, 2.0 mm/s pretest speed, 1.0 mm/s posttest speed, 1.0 mm/s test speed, 70% strain, 5 g trigger type, and a 1 s interval between two compressions.

### Low-field nuclear magnetic resonance

Transverse relaxation time (T2) of the dough samples was measured using a low-field nuclear magnetic resonance (LF-NMR) system (NMI20-015V-I, Suzhou, China). The test parameters were as follows: the number of sampling points (TD) was 139994, regulated analog gain 1 (RG1) was 10.0, the regulated first data (RFD) was 0.002, the regulated digital gain (DRG) was 3.0; the preamplifier regulate gain (PRG) was 1, and the number of sampling (NS) was 4. The T2 relaxation map was obtained after inverting the exponential attenuation curve. There were three states of water in the dough: bound water (T21), weakly bound water (T22), and free water (T23).

### Rheological properties

The samples were measured with a DHR-1 hybrid rheometer (TA Instruments, Inc., New Castle, Delaware, American) in the oscillation mode using the method described by Chen et al. (24). The samples were loaded within a 1 mm gap between two parallel plates (upper plate diameter = 30 mm). A thin layer of glycerin was applied around the sample to prevent dehydration. The samples were equilibrated at 25°C for 2 min and then heated from 25 to 100°C at a controlled heating rate (0.1°C/min). The dynamic rheological properties were determined by recording the storage modulus (G′) or elastic component (G″).

### Microscopic observation

The sample microstructures were observed with a scanning electron microscope (SEM) (SU3400, Tokyo, Japan) using the method of Yuan et al. (25). The samples were covered with a thin gold layer and immediately observed using an SEM at 1,000× magnification.

### Statistical analysis

All the data points in the experiment were acquired in triplicate. The statistical differences (*p* < 0.05) between the average values were analyzed using the Statistics 9.0 software and Tukey’s HSD (Honestly Significant Difference) comparison test.

## Results

### Determination of Mung bean proteins and hydrothermally treated Mung bean proteins gel properties

**FIGURE 1:**
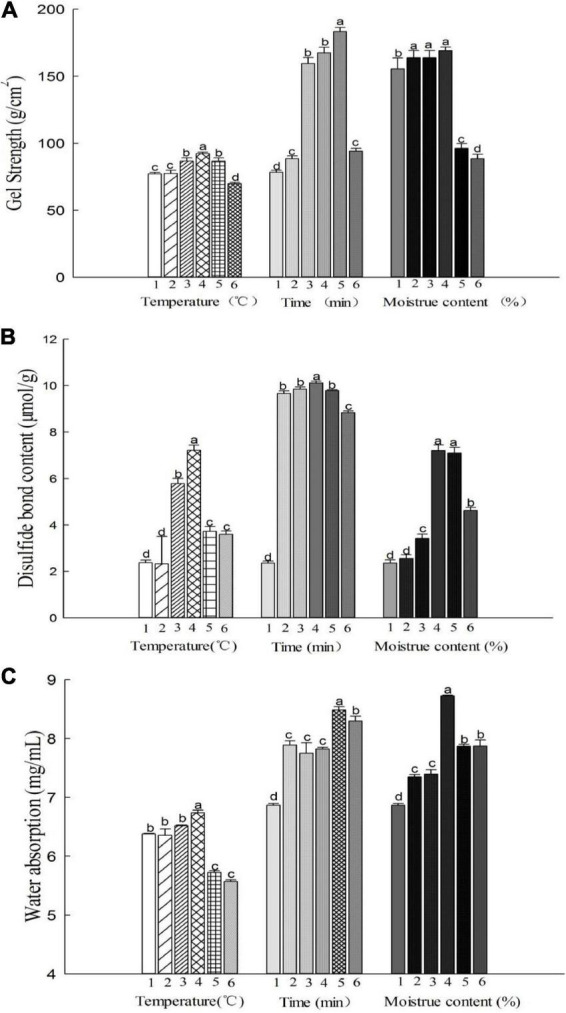
Gel properties of the MBPs under different hydrothermal treatment conditions. Numbers 1–6 for the temperature represent the MBP groups treated with different hydrothermal temperatures (25, 65, 75, 85, 95, and 105°C, respectively). Numbers 1–6 for the time represent the MBP groups treated with different hydrothermal times (0, 15, 30, 45, 60, and 75 min, respectively). Numbers 1–6 corresponding to the moisture content represent the MBP groups treated with different moisture contents (0, 15, 20, 25, 30, and 35%, respectively). The letters a–d represent significant differences between the groups. **(A)** Gel strength. **(B)** Disulfide bond content. **(C)** Water absorption.

Figure 1 depicts the effect of hydrothermal treatment on the gel strength of the MBPs at different heating temperatures (25, 65, 75, 85, 95, and 105°C), moisture contents (0, 15, 20, 25, 30, and 35%), and treatment durations (0, 15, 30, 45, 60, and 75 min). When the MBPs were heated at or above 75°C, their gel strength increased significantly and reached 97 g/cm^2^ at 85°C, which was higher than that of other treatment groups. Interestingly, when they were heated at 95 and 105°C, their gel strength decreased gradually to 90 and 74 g/cm^2^ (Figure 1A).

The disulfide bond contents had a positive correlation with the gel strength, and the gel network structure was mainly connected by disulfide bonds. Figure 1B depicts the variation in the disulfide bond content in the MBPs under different treatment conditions. The effect of heating time on the disulfide bond content varied slightly, except for the group that was treated for 75 min. However, the effect of moisture content and temperature on the disulfide bonds was particularly significant, indicating that the intermolecular interactions of the MBPs increased at an appropriate temperature and moisture content and that the number of protein molecules that favored the formation of a gel network structure increased, promoting the formation of disulfide bonds between the MBP molecules and increasing the gel strength. When the MBPs were hydrothermally treated at 85°C for 60 min with a moisture content of 25%, their disulfide bond contents and gel strength reached about 10 μmol/g and 183 g/cm^2^, respectively, which were significantly higher than those of the untreated group.

Figure 1C shows the water absorption capacity of the MBPs under different treatment conditions. There was no significant difference in the water absorption capacity of the MBPs when the temperature was lower than 85°C (*p* > 0.05); however, the water absorption capacity significantly decreased (*p* < 0.05) when the temperature was above 85°C. This may be because the denaturation temperature of the MBPs was 85°C, and the peptide chain of the MBPs unfolded as the temperature increased, resulting in a decrease in the hydration of the MBP hydrogen and ionic bonds. The water absorption capacity of the MBPs increased as the hydrothermal treatment time increased. The MBPs had the highest water absorption capacity when heated at 85°C for 60 min, but the water absorption capacity decreased when the treatment time was 75 min. The moisture content had a significant effect on the water absorption capacity of the MBPs, and this effect was notably strong when the moisture content was 25% but significantly weak when the moisture content exceeded 25%, which is consistent with the gel strength and disulfide bond content results. The above results indicate that the gel properties of the MBPs increased when treated with a 25% moisture content at 85°C for 60 min. The hydrothermal treatment caused the MBP structure to unfold and exposed the sulfhydryl groups originally buried in the center of the protein, which easily formed disulfide bonds between the protein molecules and enhanced intermolecular forces, forming a dense gel network structure and improving its water absorption capacity.

### Determination of changes in the Mung bean proteins and hydrothermally treated Mung bean proteins structures

#### Sodium dodecyl sulfate—polyacrylamide gel electrophoresis

**FIGURE 2:**
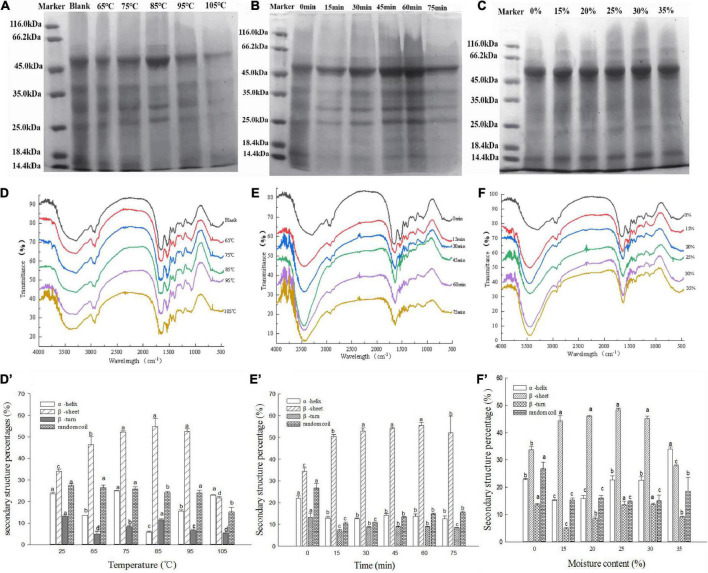
Changes in the structure of the hydrothermally treated MBPs at different controlled temperatures (25, 65, 75, 85, 95, and 105°C), processing times (0, 15, 30, 45, 60, and 75 min) and moisture contents (0, 15, 20, 25, 30, and 35%). **(A–C)** SDS–PAGE of the MBPs under the different treatment conditions, respectively. **(D–F)** FTIR spectra of the MBPs under the different treatment conditions, respectively. **(D’–F’)** Secondary structure percentages of the MBPs under different treatment conditions based on FTIR analysis. The letters a–d indicate significant differences between the groups.

SDS–PAGE was performed to investigate the effects of the hydrothermal treatment methods on the molecular weight and patterns of the MBPs (Figures 2A–C). The major protein band of the MBPs was observed at 45, 35, 30, 25, and 16 kDa. However, the protein bands of the hydrothermally treated MBPs were observed at nearly 66.2 kDa, indicating that the treated MBP produced new macromolecules. The disulfide bonds were the main force affecting gel properties; meanwhile, there were also hydrophobic bonds, hydrogen bonds, and ionic bonds. Yang et al. found that there were four kinds of forces in heat-treated soybean protein gel, which were disulfide bond, hydrophobic force, hydrogen bond, and classical force in order of magnitude (17). And some studies showed the existence of Tyr-Tyr and carbonyl-NH_2_ in proteins (26, 27). This research confirmed the presence of disulfide bonds in MBPs gels, but the difference was not significant. Moreover, FTIR results showed the existence of hydrogen bonds in MBPs. In particular, the 66.2, 45, 30, and 25 kDa protein bands of the hydrothermally treated MBPs thickened as the heating time increased at 85°C (*p* < 0.05), indicating that high temperatures cause protein degradation and internal structural damage of MBPs. However, all the protein bands of the MBPs narrowed at temperatures above 85°C and almost disappeared at 100°C. There was no significant change under different moisture conditions.

### Fourier-transform infrared spectroscopy

Figures 2D–F show changes in the MBP secondary structure observed by FTIR spectroscopy under different treatment conditions. An amide I band at 1,600–1,700 cm^–1^ was observed in all the samples. Spectral shifts at 1,600–1,700 cm^–1^ occurred in the hydrothermal temperature-treated and time-treated groups. The MBP samples treated at a temperature above 85°C for over 30 min exhibited an increase in peak intensity and a significant redshift in the amide I bands (Figures 2D,E). At the same time, the secondary structure contents (α-helix, β-sheet, and random coils) of the MBPs changed significantly (*p* < 0.05) (Figures 2D’–F’). These results indicate that the increase in temperature and duration caused the secondary structure of the MBPs to change or the peptide chains of the MBPs to rearrange. Compared to those of the temperature-treated and time-treated MBPs, the spectral shifts in the amide I band of the MBPs with different moisture contents did not change significantly (*p* > 0.05). However, spectral blue shifts and increasing peak intensities were observed at 3,250–3,500 cm^–1^ (associated with O–H stretching vibration) as the moisture content increased (particularly above 25%), and the result was similar to that of the heat-treated and time-treated groups. The hydrothermally treated MBPs were believed to have undergone changes in their original spatial structure, which disrupted intramolecular hydrogen bonds and increased intermolecular forces, thereby increasing the β-sheet content and facilitating the formation of a gel network structure.

### Noodle quality evaluation

#### Effect of modified Mung bean proteins addition on the cooking properties of noodles

**FIGURE 3:**
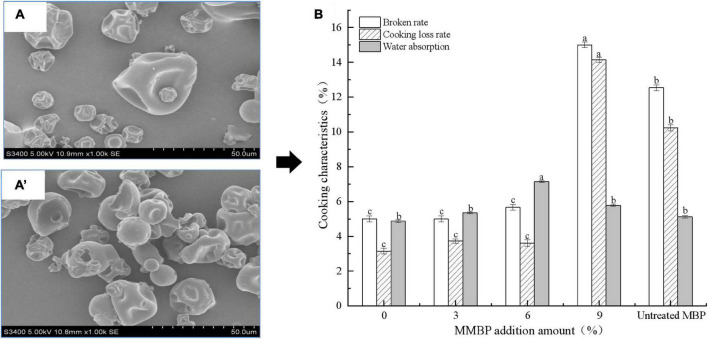
Cooking properties of noodles treated with different MMBP amounts (0, 3, 6, and 9%). **(A)** and **(A’)** Microstructure of the hydrothermally treated and untreated MBPs, respectively. **(B)** Cooking properties of the noodles. The letters a–c indicate significant differences between the groups.

Figures 3A,A’ depict the microstructures of the hydrothermally treated and untreated MBPs. The native MBPs had prominent depressed spherical particles, whereas the MMBPs had relatively large spherical expansion structures. As shown in the SEM images, the MMBP particle surfaces had larger holes, and there were noticeable cross-linked aggregates between the particles, which were different from those of the native MBPs, indicating that the ordered structure of the protein was destroyed by the hydrothermal treatment, thereby exposing hydrophobic groups and allowing protein molecules to interact with water molecules and other groups.

Figure 3B shows the effects of MMBP addition on the cooking loss rate, broken rate, and water absorption capacity of the noodles. During cooking, the cooking loss and broken rates increased as the substitution level increased, but no statistically significant differences were found between the 3 and 6% substitution levels. The broken and cooking loss rates of the noodles with 3% untreated MBPs were found to be similar to those of the 9% MMBP-treated noodle group, indicating that the MMBPs increased both the protein content and quality of the noodles. During cooking, the MMBP-treated noodles had a significantly higher water absorption capacity than the control group; however, the 9% MMBP-treated noodle group had a significantly lower water absorption than the 6% MMBP-treated noodle group, but it had a higher water absorption capacity than the control group, which may be due to strong water—starch—gluten interactions forming a highly dense structure and decreasing the water absorption capacity. Overall, the 6% MMBP-treated noodles had the best cooking characteristics.

#### Effect of modified Mung bean proteins addition on the texture properties of noodles

Among the TPA parameters of the noodle samples, gumminess, chewiness, and cohesiveness had a significant positive correlation with hardness (Table 1). Hardness depends on the degree of formation of a gluten network structure in noodles, and it plays an important role in maintaining the quality of noodles. The hardness and springiness of the cooked noodles increased linearly as the MMBP amount increased. When the MMBP amount increased to 9%, the hardness of the noodles increased, but the springiness decreased sharply due to the decrease in water absorption capacity of the high-protein noodles (Figure 3B).

**TABLE 1:** Texture properties of the noodles treated with different MMBP amounts.

Addition (%)	Hardness (g)	Springiness(%)	Cohesiveness (g^⋅^s)	Gumminess (g)	Chewiness (g^⋅^s)
0	11629.13 ± 150^d^	0.64 ± 0.05^c^	0.61 ± 0.01^d^	7845.27 ± 143^d^	6640.68 ± 125^a^
3	13778.32 ± 154^c^	0.67 ± 0.02^b^	0.63 ± 0.03^c^	9717.01 ± 103^c^	6567.32 ± 132^a^
6	15553.59 ± 134^b^	0.74 ± 0.04^a^	0.65 ± 0.02^b^	10507.60 ± 201^b^	6369.66 ± 132^b^
9	16661.92 ± 121^a^	0.68 ± 0.02^b^	0.69 ± 0.02^a^	12442.68 ± 209^a^	6062.90 ± 197^c^

^a–d^ Indicates significant differences between the groups.

#### Effect of modified Mung bean proteins addition on the water distribution of wheat dough

**FIGURE 4:**
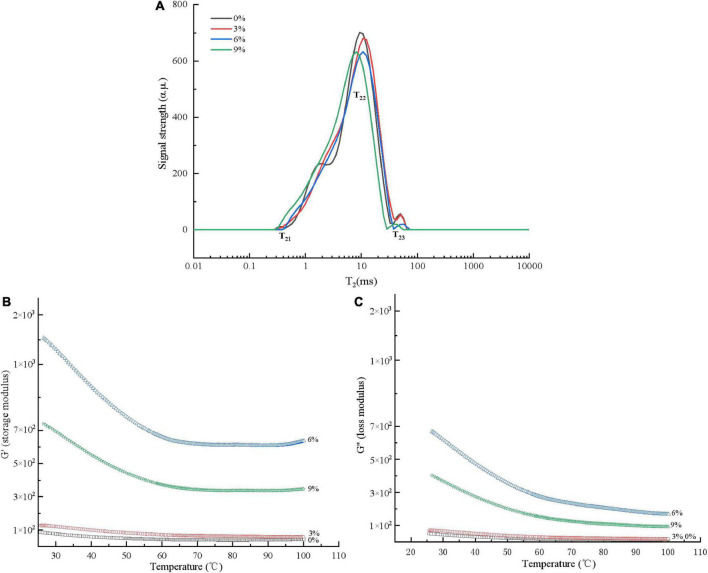
Water distribution and rheological properties of the dough treated with different MMBP amounts (0, 3, 6, and 9%). **(A)** Water distribution, **(B)** storage modulus, and **(C)** loss modulus.

Moisture plays an important role in noodle processing, and the macro quality characteristics of noodles (such as texture) are mostly determined by the water absorption capacity and internal moisture state of the dough. The MMBP-treated wheat dough had a slightly shorter relaxation time (T21, T22, and T23) than the wheat dough group (Figure 4A), indicating that the water molecules and macromolecules such as protein and starch were closely integrated. The relaxation time of the different MMBP-treated doughs shifted to the left as the added MMBP amount increased, indicating that the high-protein dough had a denser and stronger ability to bind water than the low-protein dough. This also indicated that the compact gluten network inhibited the swelling of the noodles, thereby affecting the dry matter water absorption capacity of the noodles, which is consistent with the findings on the edible properties of the noodles (Figure 3B).

#### Effect of modified Mung bean proteins addition on the rheological properties of wheat dough

Figures 4B,C depict the rheological properties of MMBP-treated wheat dough. Using MMBPs to partially replace wheat resulted in a noticeable increase in G′ and G″ values. Additionally, G′ was higher than G″ for all the groups, indicating that the dough was more elastic than sticky. The 6% MMBP-treated group had the highest G′ and G″ values among the MMBP-treated groups, indicating that the MMBP-treated dough had an increased resistance to deformation and that the MMBPs cross-linked with the wheat proteins to form a network structure. However, compared to those of the 6% MMBP-treated group, the G′ and G″ values of the 9% MMBP-treated group significantly decreased, reaching 53 and 50%, respectively. The rheological results at different temperatures also revealed that the MMBPs increased the G′ and G″ values of the dough, which is consistent with the texture property results.

#### Effect of modified Mung bean proteins addition on the wheat dough microstructure

**FIGURE 5:**
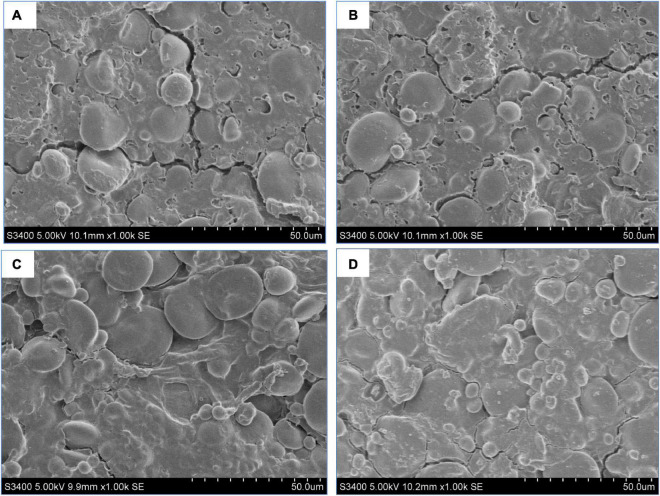
Optical microscopic images of the microstructures of the dough treated with different MMBP amounts (0, 3, 6, and 9%). Original magnification: 1000x. **(A)** Wheat dough, **(B)** 3% MMBP-treated dough, **(C)** 6% MMBP-treated dough, and **(D)** 9% MMBP-treated dough.

Figure 5 shows the microstructure of the dough network treated with MMBPs. Starch granules were wrapped or embedded in the dough gluten protein, forming a relatively dense and uniform network structure, but some cracks were observed in the network structure (Figure 5A). The MMBPs were able to repair the dough cracks. The 3% MMBP-treated dough group had a denser network structure and smaller cracks than the untreated dough group (Figure 5B). The 6% MMBP-treated dough group had a relatively dense gluten network structure, and their cracks disappeared completely (Figure 5C). The 9% MMBP-treated dough group had the most compact network structure, with starch particles tightly wrapped (Figure 5D). These results confirm that MMBPs improve the ability of doughs to form stabilized cross-linked structures. However, the 9% MMBP-treated dough group had lower textural properties than the other groups, which may be because MMBP altered the force of gluten proteins (Figure 5D).

## Discussion

MBPs are excellent plant-derived proteins that can provide nutritional supplements to a grain-based diet. However, their use in staple foods is limited because they lack gluten. Current research has found that improved protein gelation can broaden the application of MBPs in food products such as sausages, tofu, and noodles. In this study, hydrothermal treatment (temperature, time, and moisture content) was found to significantly affect the gel properties of MBPs because it disrupts the MBP structure, thereby exposing hydrophobic groups and intensifying protein interactions (28, 29). Chunkao et al. demonstrated that 85°C is the denaturation temperature of MBPs and that denatured MBPs expose more hydrophobic regions and sulfhydryl groups, resulting in high gel strength (30). The formation to stabilization stages of the MBP gels were discovered to occur at 85°C for 0–60 min. However, prolonged high-temperature treatment of MBPs causes dissociation of protein–peptide chains, which decreases gel strength. Yang et al. confirmed that soybean protein subjected to prolonged high temperature treatment leads to complete denaturation and also hydrolysis of protein to produce peptide chains or free amino acids, and these small molecular units cause a reduction in gel strength (17). MBPs with more than 25% moisture contents disrupted the intermolecular and protein—water binding capacities, affecting gel formation and maintenance. The increase in protein moisture contents caused a decrease in protein denaturation temperature due to the critical influence of hydration and water permeation in the protein molecular conformation. Wang et al. demonstrated that the water molecules of proteins with high moisture content partially penetrate into the surface of the protein structure, causing the protein to swell, thereby changing the flexibility of the protein-peptide chains and making it easier to form peptide hydrogen bonds (18). This result is consistent with the water absorption capacity results of the MBPs. In this study, the gel strength had a positive correlation with the disulfide bond content because the denatured MBPs were randomly aggregated to form disulfide bonds through intermolecular hydrophobic interactions, resulting in the cross-linking, folding, and rearrangement of the spatial protein structure (31). The above results suggest that the covalent polymerization of MBP molecules enhances their gelation properties. The SDS–PAGE and FTIR results confirmed this hypothesis. The SDS–PAGE analysis revealed that the hydrothermally treated MBPs produced a new protein band near 66.2 kDa, which is similar to previous findings (9). However, prolonged high temperatures thinned the protein bands, indicating that the MBP peptide chains degraded, resulting in decreased gel strength. The FTIR analysis results revealed that the treated MBPs had a significantly higher β-sheet content than the untreated MBPs (Figure 2). Protein aggregation had a positive correlation with the β-sheet content, and high β-sheet contents facilitated the formation of gel networks (32). The microstructural changes confirm that the treated MBP structure was altered, which improved its functional properties.

Recent studies have demonstrated that adding proteins to noodles is an effective strategy for improving their nutritional value. However, it can also cause a decrease in noodle quality. In this study, the broken rate of the 3% untreated MBP noodles reached about 13.21% (5.02% for wheat noodles). The MMBP-treated noodles did not have different cooking characteristics from wheat noodles. When 9% of MMBPs was added, there was a significant difference because adequate amounts of MMBPs could cross-link with wheat dough and enhance the gluten network structure, favoring the noodle steaming characteristics. However, excess MMBPs were further cross-linked with the wheat dough, resulting in increased hardness and decreased water absorption capacity, which is consistent with the texture property results (Table 1). The changes in moisture distribution, rheological properties, and microstructure of the MMBP-treated dough indicated the cross-linking reaction between the MMBPs and wheat dough. The relaxation time of the MMBP-treated dough shifted to the left, indicating that the water in the dough changed from weakly bound water to strongly bound water under the forces of hydrogen bonding, hydrophobic interaction, and van der Waals forces with the MMBPs and wheat proteins (33). The 9% MMBP-treated dough had the strongest bound water, indicating that the changes in the forces between the MMBPs, wheat proteins, and water molecules affected the gluten network structure of the dough, decreasing the cooking characteristics of the noodles (34–36). Strong cross-linking between molecules in mixed dough increases the molecular mass of polymers in the dough, thereby increasing the G′ and G″ values, which is consistent with the findings of this study. The microstructure results confirmed the strong cross-linking reaction between the MMBPs and wheat dough, which resulted in a dense gluten network structure in the MMBP-treated dough (Figure 5).

## Conclusion

In conclusion, these results indicate that MMBPs, as a type of foreign protein, promoted an exchange reaction between sulfhydryl and disulfide bonds in the dough, increased the probability of protein–peptide chain cross-linking, folding, and rearrangement, and enhanced the ability of the protein to wrap starch particles, resulting in a more compact dough and a strengthened internal network structure.

## Data availability statement

The original contributions presented in this study are included in the article/supplementary material, further inquiries can be directed to the corresponding authors.

## Author contributions

JD designed the study, interpreted the results, and drafted the manuscript. YT performed the analysis. HC collected the test data. DZ and CW modified the manuscript. All authors contributed to the article and approved the submitted version.
